# Research priorities for intensive care: A stakeholder-informed priority-setting partnership

**DOI:** 10.1016/j.ccrj.2026.100215

**Published:** 2026-09-11

**Authors:** Samantha Keogh, Andrew J. Neilson, Annabel Levido, Antony Attokaran, Amanda Corley, Felicity Edwards, Kevin B. Laupland, Stephen Luke, Prashanti Marella, Andrea Marshall, Philippa McIlroy, Dale Trevor, Mahesh Ramanan, the Queensland Critical Care Research Network (QCCRN), Julia Affleck, Antony G. Attokaran, Stuart Baker, Neeraj Bhadange, Jane Brailsford, Karen Carson, Georgia Clark, Pierre Clement, George Cornmell, Samantha Dash, Kerina J. Denny, Jayesh Dhanani, Pam Dipplesman, Ra'eesa Doola, Felicity Edwards, Janine Garrett, Peter Garrett, Denzil Gill, Michelle Gatton, Jatinder Grewal, Rod Hurford, Kylie Jacobs, Nermin Karamujic, Samantha Keogh, Kiran Shekar, Aashish Kumar, Vijo Kuruvilla, Kevin B. Laupland, Jayshree Lavana, Paula Lister, Stephen Luke, Langa Lutshaba, Josephine Mackay, Andrea Marshall, Prashanti Marella, Vikram Masurkar, Craig McDonald, James McCullough, Jason Meyer, Lynette Morrison, Lauren Murray, Ahmad Nasser, Ben Nash, Alex Nesbitt, Anni Paasilahti, Dinesh Parmar, Kunjal Patel, Hamish Pollock, Jaco Poggenpoel, Raju Pusapati, Lachlan Quick, Eamon Raith, Mahesh Ramanan, Gul E. Rana, Josephine Reoch, Sunil Sane, Siva Senthuran, Vikram Shah, Judy Smith, David Stewart, Alexis Tabah, Cath Tacon, Jennifer Taylor, Mandy Tallott, Zephanie Tyack, Karthik Venkatesh, Adam Visser, James Walsham, Monica Watson, Stephen Whebell, Kyle White, Emma Williams, Patrick Young

**Affiliations:** a School of Nursing and Centre for Healthcare Transformation, Queensland University of Technology, Brisbane QLD, Australia; b Intensive Care Services, Royal Brisbane and Women's Hospital, Brisbane QLD, Australia; c Nursing and Midwifery Research Centre, Royal Brisbane and Women's Hospital, Brisbane QLD, Australia; d School of Nursing and Midwifery, University of Queensland, Brisbane QLD, Australia; e Intensive Care Unit, Rockhampton Hospital, QLD, Australia; f Mayne Academy of Critical Care, Faculty of Medicine, The University of Queensland, Brisbane, QLD, Australia; g School of Nursing and Midwifery, Griffith University, Brisbane QLD, Australia; h School of Medicine, Queensland University of Technology, Brisbane QLD, Australia; i Intensive Care Unit, Mackay Hospital and Health Service, Mackay QLD, Australia; j Department of Intensive Care Medicine, Caboolture Hospital, QLD, Australia; k Nursing and Midwifery Education and Research Unit, Gold Coast University Hospital, QLD, Australia; l Intensive Care Unit, Cairns Hospital, Brisbane, QLD Australia; m Consumer and Community Network, Royal Brisbane and Women's Hospital, QLD, Australia; n Critical Care Division, The George Institute for Global Health, University of New South Wales, Sydney NSW, Australia

**Keywords:** Consumer engagement, Health services research, Intensive care, Modified James Lind Alliance, Research prioritisation

## Abstract

**Objective:**

The aim of the present study was to identify and prioritise unanswered research questions in intensive care through a stakeholder-driven priority-setting partnership involving clinicians and consumers.

**Design:**

Structured collaborative consultation using a modified James Lind Alliance Priority Setting Partnership approach. The process included five stages: initiation, consultation, collation, interim prioritisation, and a final consensus workshop. Submissions were collated into indicative questions, refined and shortlisted by the study team, and ranked in an interim prioritisation survey. Final priorities were determined through an in-person workshop using a modified nominal group technique.

**Setting:**

Queensland Critical Care Research Network, a statewide intensive care research network in Queensland, Australia.

**Participants:**

Clinicians working in intensive care and consumers with lived experience of intensive care as patients or family members.

**Interventions:**

Not applicable.

**Results:**

Fifty-five participants submitted 136 research questions, which were consolidated into 76 indicative questions and subsequently shortlisted to 30 for interim prioritisation. Eighty-three participants completed the ranking survey. Sixteen stakeholders (clinicians and consumers) participated in the final workshop, resulting in consensus on 12 priority research questions. Key priorities included clinical and technology-enabled decision-making, survivorship and recovery, workforce and systems of care, and delirium prevention. Consumer partnership and implementation were identified as overarching principles underpinning future research.

**Conclusions:**

These priorities provide a stakeholder-informed foundation for future intensive care research, supporting collaborative research programs, funding strategies, and meaningful researcher–clinician–consumer partnerships.

## 1 Introduction

Critical illness is associated with significant mortality, long-term morbidity, and high healthcare resource use.^1^^,^^2^ Despite a large and growing evidence base, important uncertainties remain in many areas of intensive care practice, and translation of findings is variable.

Increasingly, there is recognition that research priorities should reflect the needs of those who deliver and experience care. However, in intensive care, research agendas have traditionally been driven by academic, clinical, or industry interests, with limited structured involvement of patients in identifying areas of priority investigation.^3^^,^^4^ In response, there has been a growing international movement to embed meaningful consumer and clinician involvement throughout the research lifecycle. In Australia, this shift is reflected in the National Clinical Trials Governance Framework and National Health and Medical Research Council guidance, which emphasise the importance of consumer partnership from agenda setting through to implementation.^5^^,^^6^

Priority-setting partnerships provide a structured approach to identifying and prioritising unanswered research questions through collaboration with clinicians and consumers. The James Lind Alliance (JLA) framework^7^ has become the most widely adopted methodology for health research priority setting internationally and has been applied across numerous clinical fields.[8], [9], [10] Within critical care, JLA Priority Stetting Partnerships – formal and modified – have recently been completed or initiated in the United Kingdom^11^ and Canada,^12^ and within specific populations such as sepsis,^13^^,^^14^ and paediatric critical care in Australia.^15^^,^^16^ However, research priorities are inherently influenced by the demographic, geographical, organisational, and health-system context in which care is delivered. Consequently, priorities identified in one jurisdiction may not fully reflect the needs, challenges, and opportunities of another. To our knowledge, no previous JLA Priority Setting Partnership had established multidisciplinary adult intensive care research priorities across a Queensland clinical network. Establishing priorities at the network level may support the development of contextually relevant collaborative research programs and inform future funding strategies.

The Queensland Critical Care Research Network (QCCRN) provided an established platform for multidisciplinary collaboration across statewide intensive care services.[17], [18], [19], [20] Identifying shared priorities within this network offered an opportunity to align future research activity within areas of greatest perceived need and potential impact.

The aim of this study was to identify and prioritise the most important unanswered research questions in intensive care, as determined by clinicians and consumers, using a modified JLA priority setting approach.

## 2 Method

### 2.1 Study design

A modified JLA Priority Setting Partnership approach was used to identify and prioritise unanswered research questions in contemporary intensive care. The study was designed to coproduce a research agenda aligned with end-user needs. The project followed core JLA principles of transparency, inclusivity, and iterative consensus building, while adapting procedures to accommodate a Queensland-wide stakeholder group.^7^

This study aligns with recommendations for reporting patient and public involvement in research (REPRISE).^21^

### 2.2 Setting and overall process

**Fig. 1:**
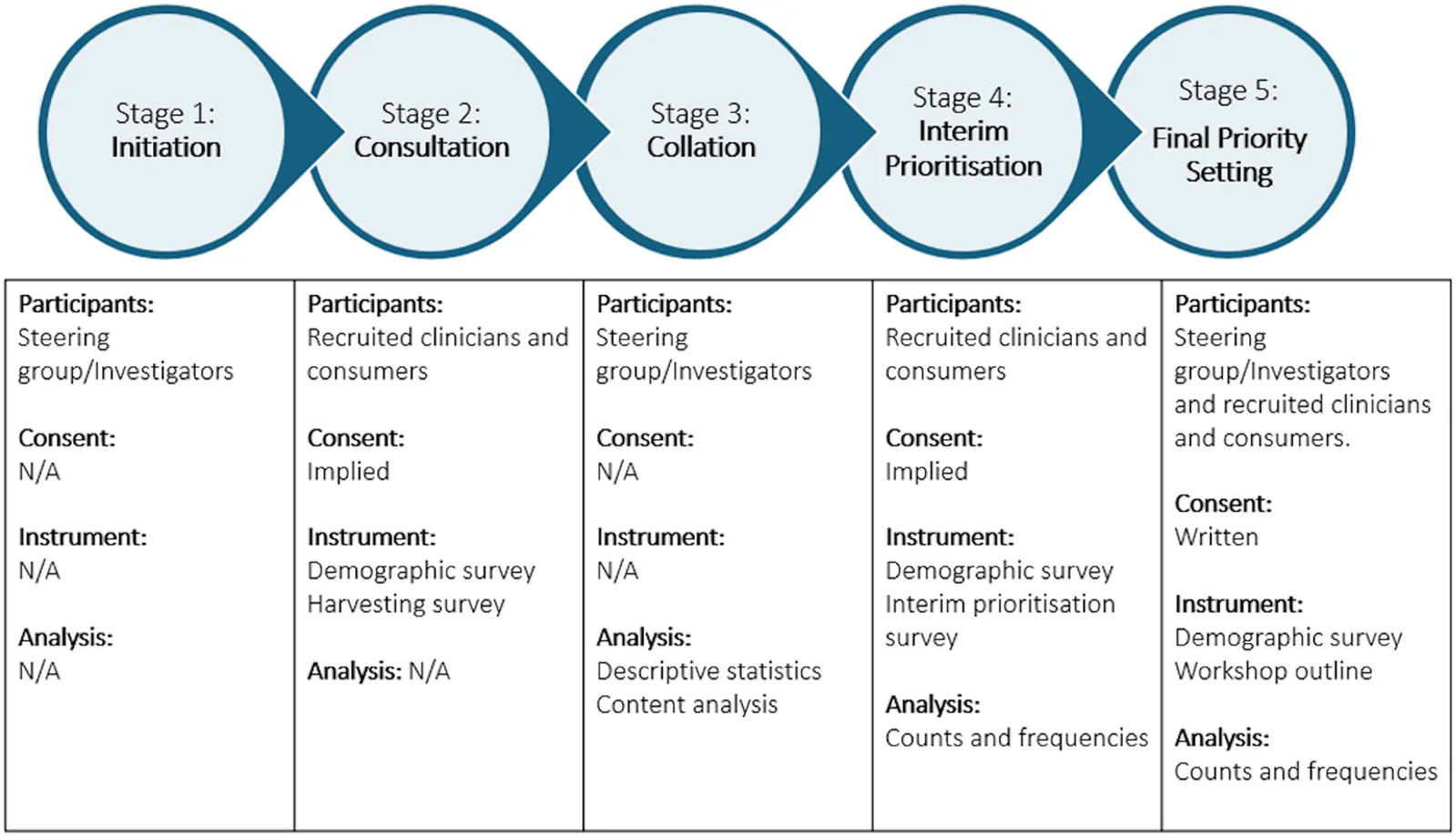
Five key stages of the research prioritisation process.

The priority-setting process comprised five sequential stages (see Fig. 1):

1. Initiation: establishment of the steering group and study scope
2. Consultation: harvesting survey
3. Collation and development of indicative questions
4. Interim prioritisation: ranking survey
5. Final priority-setting workshop

Electronic surveys were administered using Research Electronic Data Capture (REDCap) hosted by Queensland University of Technology (QUT).^22^^,^^23^ The final consensus workshop was conducted in person using a modified nominal group technique consistent with the JLA approach.^7^

### 2.3 Participants and recruitment

Participants included clinicians and consumers with recent experience of the intensive care environment. Specifically:

• a consumer (patients or family members with intensive care unit [ICU] experience) or
• a clinician working in or closely with intensive care.

Clinicians could include nursing, medical, allied health, education, or research professionals.

Participants were recruited through the QCCRN, a statewide multidisciplinary network of intensive care clinicians and researchers. Recruitment occurred via email distribution lists using a snowball sampling approach, whereby participants were encouraged to share the study invitation within their professional and consumer networks to optimise reach. Targeted recruitment was subsequently undertaken to improve the representation of underrepresented groups.

Participation was voluntary and anonymous, with completion of the survey implying consent. Full informed consent was obtained from participants attending the final workshop. The study was approved by the QUT Human Research Ethics Committee (ref: 10092). Data were stored on secure, password-protected QUT servers.

The study was conducted between October 2025 and March 2026. Question harvesting occurred between October and December 2025, interim prioritisation was undertaken in January and February 2026, and the final consensus workshop was conducted in March 2026.

### 2.4 Stage 1: Initiation

A steering group comprising key stakeholders, including clinicians, consumer representatives, and clinician researchers, was established to guide the project. During the initial meeting, the group defined the project scope and developed a harvesting survey to obtain input from ICU end-users on potential research priorities.

### 2.5 Stage 2: Consultation

A harvesting survey invited clinicians and consumers to submit important unanswered research questions in intensive care. Participants were asked to identify priority topic areas and suggest research questions based on their experience. Demographic data were collected to assess the representation of clinician and consumer participants and to inform targeted recruitment if required.

The consultation survey also included an optional open-ended question inviting participants to suggest strategies to improve participation in intensive care research. Responses to this question were analysed using conventional content analysis. Two investigators independently reviewed responses, developed preliminary codes, and (through discussion and consensus) grouped codes into overarching categories. These findings were summarised descriptively, with detailed results reported in the Supplementary material.

### 2.6 Stage 3: Collation

Free-text responses were independently reviewed and descriptively coded. Similar submissions were grouped into topic clusters through consensus discussion. Topics were considered conceptually saturated when multiple responses reflected the same underlying uncertainty without introducing new populations, interventions, comparators, or outcomes. These were consolidated into a single indicative research question to preserve the respondent intent while avoiding duplication. No topics were excluded based on frequency alone. Questions outside the scope of intensive care, those too broad to represent a researchable uncertainty, or those clearly addressed by existing evidence were removed or reframed.

Consistent with JLA guidance, the list generated during collation was subjected to an iterative consolidation and evidence-checking process undertaken by a core study group (SK, AL, AJN, AC, AM, MR) to produce a manageable list for interim prioritisation. In keeping with the JLA methodology, this process was conducted independently by members of the study group, followed by group comparison and discussion of consolidated questions to resolve differences and reach consensus. Questions were assessed against the following criteria:

• Does the question represent a clear area of uncertainty (i.e. is it not already adequately answered by existing evidence)?
• Is the question distinct and nonduplicative?
• Is the wording understandable to a broad stakeholder audience?

Conceptually similar questions were merged and reframed as broader “research direction” questions consistent with JLA guidance. Where appropriate, closely related questions were separated to preserve distinct uncertainties raised by respondents.

### 2.7 Stage 4: Interim prioritisation survey

The consolidated research questions were circulated in a second digitally administered survey using the REDCap external module framework.^24^ Participants were asked to select and rank the ten questions they considered most important for future research, ordering them from highest to lowest priority. Responses were scored according to each participant’s ranking, with the highest priority assigned ten points and the lowest one point.

### 2.8 Stage 5: Final priority-setting workshop

A one-day in-person workshop was conducted using an adapted nominal group technique consistent with the JLA approach. Clinicians and consumers participated in facilitated small-group discussions to review interim prioritisation results and agree on the final ranked list of research priorities through iterative discussion and consensus.

## 3 Results

**Fig. 2:**
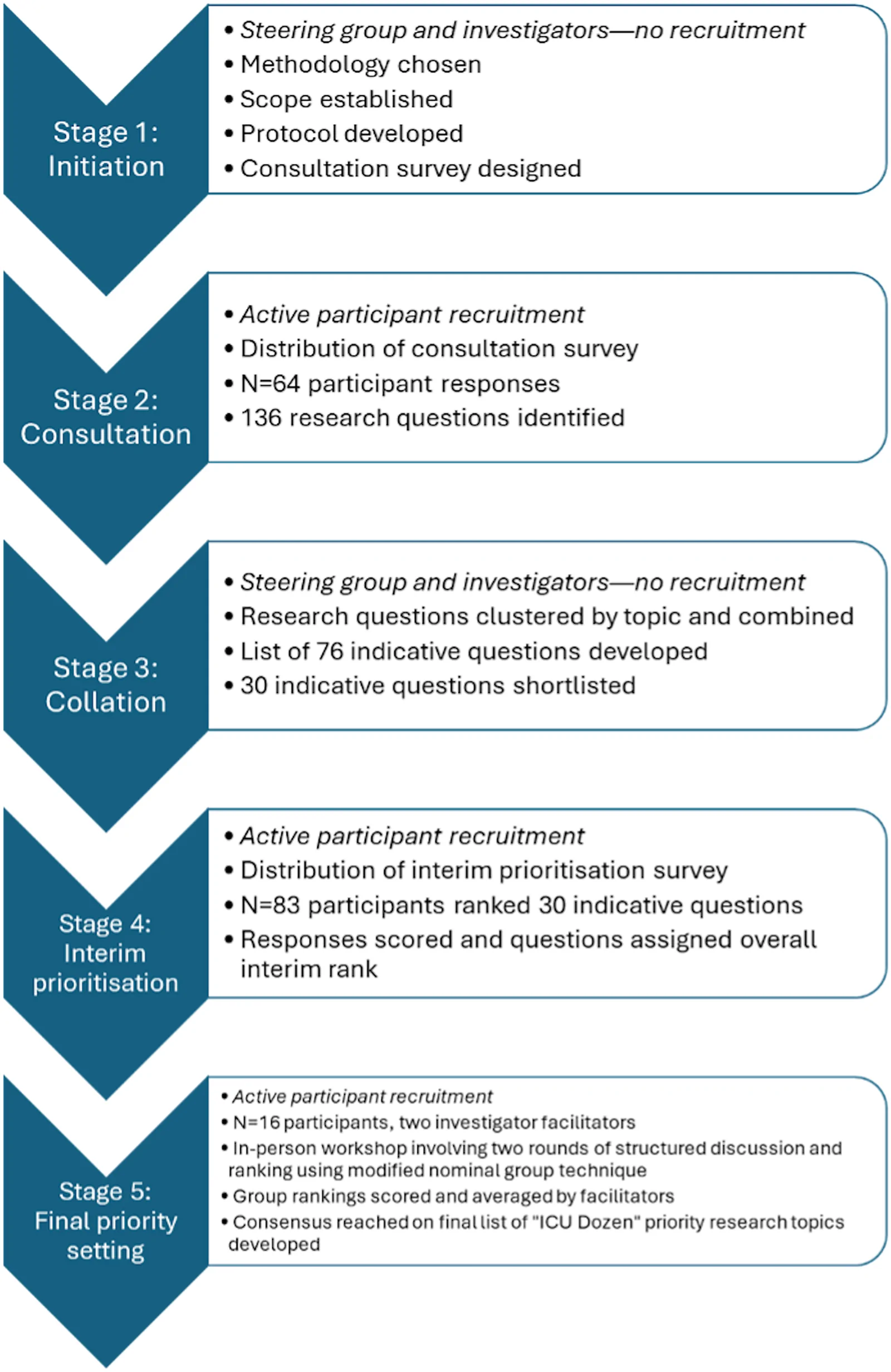
Overview of the priority-setting process and reduction of research questions.

Results of the priority-setting process are summarised in Fig. 2, including participant numbers and question reduction across each stage.

### 3.1 Stage 2: Consultation survey

#### 3.1.1 Participant demographics

A total of 150 individuals accessed the consultation survey. Fifty-nine responses contained no survey responses and 27 included demographic data only. Sixty-four responses included a mixture of broad research topics and research questions. Respondents included both clinicians (60/64, 94%) and consumers (4/64, 6%) with recent experience of intensive care.

The consumer cohort included one current consumer, one previous consumer, one partner/support person of a current consumer, and one partner/support person of a previous consumer.

Clinician participants (n = 60) included nurses (21, 33%), medical practitioners (17, 27%), allied health professionals (9, 14%; four physiotherapists, one dietitian, and four unspecified), clinician researchers (5, 8%), and one researcher (2%). A third of clinicians (22, 34%) had more than 15 y of experience; the remainder reported 1–5 y (12, 19%), 6–10 y (11, 17%), or 11–15 y of experience (8, 13%). Seven clinicians (11%) did not provide demographic information.

**Fig. 3:**
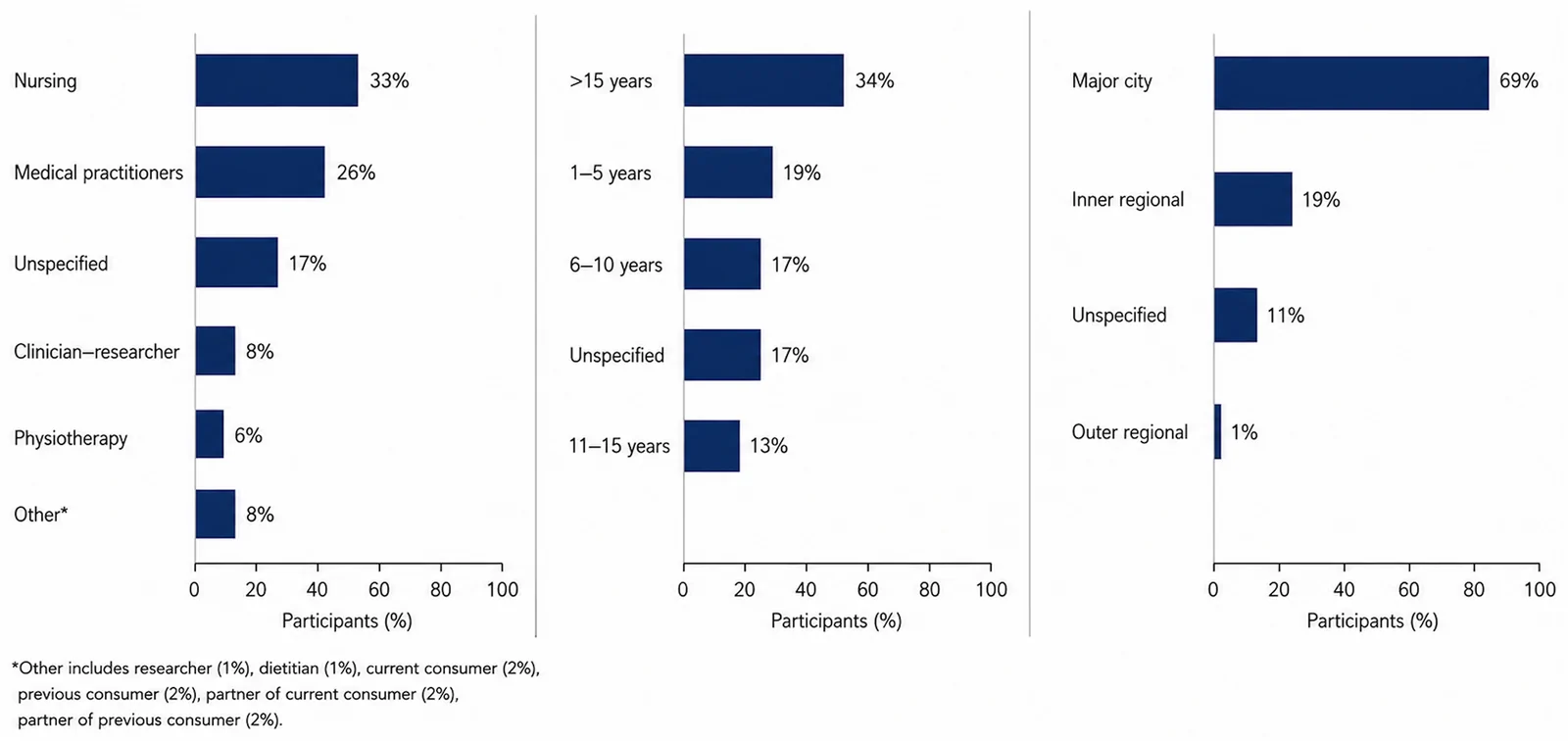
Distribution of participant profession, experience, and regionality.

Fifty-seven participants provided further demographics, reporting experience in adult ICU, while six (10%) of the clinicians also reported experience in paediatric ICU. Most (44, 77%) participants lived in a major city of Australia, while others lived in inner (12, 21%) or outer (1, 2%) regional Australia. Participants mostly identified as female (35, 61%). No participants reported identifying as Aboriginal or Torres Strait Islander (see Fig. 3).

#### 3.1.2 Harvesting of research questions

From the 55 participants who submitted research questions, 136 potential research questions were identified. Submissions covered a wide range of clinical, organisational, and recovery-focused topics.

### 3.2 Stage 3: Collation

Free-text submissions were collated into indicative research questions and organised into broader thematic domains. Following collation and refinement, 76 indicative research questions remained. The 76 indicative research questions were subsequently reduced through team consensus to a shortlist suitable for interim prioritisation consistent with the JLA approach. This process resulted in a final shortlist of 30 research questions across multiple topic domains (Supplementary File 1).

### 3.3 Stage 4: Interim prioritisation survey

A total of 83 participants completed the interim prioritisation survey, including 57 clinicians and 3 consumers, with 23 participants not providing demographic information. The final ranked questions and scores are shown in Supplementary File 2.

### 3.4 Stage 5: Final priority-setting workshop

Sixteen participants from metropolitan and regional locations across Queensland attended the final prioritisation workshop. Participants included three consumer representatives plus 11 clinicians from nursing, medicine, and allied health. Two independent and nonvoting facilitators guided the workshop. Consumer participants were paid an honorarium in accordance with Health Consumers Queensland Schedule to recognise their time and contribution.

Participants were allocated to mixed stakeholder groups and completed two rounds of structured discussion and ranking. In the first round, groups reviewed the shortlisted questions, organised them by relative priority, and ranked their top ten. Rankings were scored by facilitators using the same approach as the interim survey and aggregated to generate an interim ordering. Participants were then reallocated into new groups and the process repeated, allowing reconsideration of priorities following broader discussion.

Through this iterative process of discussion, ranking, and refinement, consensus was reached on 12 priority research questions (“the ICU Dozen”), presented in Table 1. Two overarching principles were also identified as important foundations for future research: (1) consumer partnership and codesign, and (2) implementation in clinical practice.

**Table 1:** Final ICU research priorities (n = 12).

Rank	Question
1	How can digital technologies and decision-support tools (including artificial intelligence) be used safely and effectively to improve care and workflow in intensive care?
2	How should clinicians decide when to start, continue, or stop ICU treatments and life-sustaining therapies?
3	What follow-up services and models of care best support recovery and wellbeing for ICU survivors and their families?
4	How should advanced life-support technologies (such as ECMO and ECPR) be used to improve survival and meaningful recovery in critical illness?
5	What workforce and leadership strategies best support high-performing and sustainable ICU teams?
6	How should monitoring, tests, and imaging be used to guide treatment decisions and avoid unnecessary harm?
7	What strategies best prevent and manage delirium and sleep disruption in critically ill adults while maintaining patient safety?
8	What strategies best optimise neurological care, prognosis, and recovery for critically ill patients with acute brain injury or hypoxic events?
9	What metabolic and muscle-preserving therapies best prevent ICU-acquired weakness and improve recovery after critical illness?
10	What are the long-term physical, cognitive, and quality-of-life outcomes after critical illness, and how can these be prevented or improved?
11	How can shared decision-making and goals-of-care discussions be best supported in ICU?
12	What communication approaches best support ICU patients and families during critical illness?

ICU, intensive care unit; ECMO, extracorporeal membrane oxygenation; ECPR, extracorporeal cardiopulmonary resuscitation.

Following completion of the priority setting process, the final priorities were fed back to stakeholders through circulation of the draft manuscript to the network and consumer representatives, and presentation at the network’s Annual Scientific Meeting.

### 3.5 Strategies to enhance research participation

Forty-nine participants provided suggestions to improve participation in intensive care research. Content analysis identified four key themes: communication and awareness, ease of research participation and conduct, consumer engagement, and organisational support (Supplementary Table S3). Participants highlighted the need for greater visibility of research within clinical environments, alongside simplified and streamlined processes to reduce the burden of ethics, governance, and recruitment. Meaningful consumer engagement, including accessible and human-centred approaches, was also emphasised. Organisational support, particularly through protected time, dedicated roles, and leadership endorsement, was identified as critical to enabling sustained research involvement.

## 4 Discussion

This study identified a broad and clinically relevant set of research priorities in intensive care, reflecting uncertainties that extend beyond traditional physiological interventions to include recovery and survivorship, workforce and wellbeing, systems of care, decision-making, and the increasing role of digital technologies in clinical care. This breadth reflects the evolving scope of intensive care practice and highlights where evidence remains limited, despite high clinical importance.

The prioritised questions align with emerging recognition that outcomes of critical illness extend well beyond short-term survival, with increasing emphasis on long-term recovery, quality of life, and survivorship.^1^^,^^25^ Priorities related to follow up services, neurological recovery, delirium, and ICU-acquired weakness reinforce the need to address survivorship and functional outcomes alongside acute care delivery. Similarly, the inclusion of workforce, leadership, and decision-making priorities reflects the growing acknowledgement that staffing and system-level factors influence patient outcomes in intensive care.^26^^,^^27^

Questions related to digital technologies and decision support tools emerged as a notable theme and likely reflect both increasing reliance on data-driven systems, alongside uncertainty regarding their safe and effective implementation. This finding is consistent with broader healthcare trends towards digital transformation and highlights the need for rigorous evaluation to ensure these technologies improve, rather than complicate, clinical care.[28], [29], [30]

These findings are broadly consistent with recently published intensive care Priority Setting Partnerships undertaken in Canada and the United Kingdom (UK), which similarly identified survivorship, rehabilitation, communication, family-centred care, delirium, workforce wellbeing, and implementation of evidence into practice as major research priorities.^11^^,^^12^ This convergence suggests considerable international agreement regarding many of the most important unanswered questions in intensive care. However, differences in emphasis were also evident. Compared with the Canadian and UK partnerships, Queensland stakeholders assigned greater priority to digital technologies and artificial intelligence, use of advanced life-support treatment, neurological critical care, and the optimisation of monitoring and diagnostic testing. These differences likely reflect variation in stakeholder perspectives, healthcare systems, and the demographic, geographical, and organisational context in which intensive care is delivered. This reinforces that priority setting should remain responsive to local health-system needs while contributing to an emerging international research agenda.

A key strength of this study is the use of a transparent, structured, and iterative approach to priority setting, incorporating input from clinicians and consumers culminating in a consensus workshop.^7^ The inclusion of multidisciplinary stakeholders and repeated small-group deliberations supported considerations of diverse perspectives and facilitated the development of shared priorities. However, demographic information was not provided by all survey respondents, limiting interpretation of participation representation.

Consumer participation in the survey phases was limited, which is a recognised challenge in priority-setting and health research engagement more broadly.^31^^,^^32^ This may reflect the complexity and acuity of intensive care experiences, which can limit opportunities for engagement.^33^ To mitigate this, consumer-derived questions were retained throughout the process, and consumer representatives actively participated in the final workshop. Nevertheless, the findings should be interpreted with consideration of relatively greater influence of clinician perspectives.

Additional limitations include the use of voluntary participation, which may have introduced selection bias, and the use of the modified JLA approach, whereby shortlisting was undertaken by the study team rather than a broader stakeholder group. These adaptations were necessary to ensure feasibility within a geographically dispersed network and consistent with approaches in similar studies.^8^^,^^10^^,^^34^

In addition to identifying research priorities, participants highlighted the practical considerations for improving research participation and delivery. Key themes included the importance of effective communication and awareness, simplifying participation processes, strengthening consumer engagement, and enhancing organisational support for research. These findings reinforce that identifying priorities alone is insufficient; successful translation into research activity requires attention to the structural and operational barriers that influence participation and implementation. Addressing these factors may support more effective delivery of future research aligned with the identified priorities.^33^^,^^35^

The prioritised research questions provide a pragmatic and stakeholder-informed roadmap for future intensive care research. These priorities provide a stakeholder-informed framework to guide future intensive care research, supporting collaborative research programs and funding applications. Future work should examine how these priorities align with existing research activity, funded programs, and ongoing clinical studies to identify areas of convergence, evidence gaps, and opportunities for future investment. The identification of consumer partnership and implementation as cross cutting principles further emphasise the importance of ensuring research is both meaningful and translatable in practice.

## 5 Conclusion

This modified priority-setting partnership identified a consensus set of key unanswered research questions in intensive care. The priorities provide a foundation to guide future research and collaborations, aligned with the priorities of clinicians and consumers.

## Author contributions

SK, AL, AC, AM and MR conceived and designed the study. SK, AN, AL, AC, FE, KBL, AM, DT and MR contributed to study conduct, data synthesis and interpretation. AA, SL, PM and PMcI contributed through the study steering committee and to refinement and interpretation of the research priorities. SK drafted the manuscript, and all authors critically reviewed and approved the final manuscript. The QCCRN supported stakeholder engagement and delivery of the priority-setting process.

## Collaborators

Queensland Critical Care Research Network Group (QCCRN) in alphabetical order: Julia Affleck, Antony G. Attokaran, Stuart Baker, Neeraj Bhadange, Jane Brailsford, Karen Carson, Georgia Clark, Pierre Clement, George Cornmell, Samantha Dash, Kerina J Denny, Jayesh Dhanani, Pam Dipplesman, Ra’eesa Doola, Felicity Edwards, Janine Garrett, Peter Garrett, Denzil Gill, Michelle Gatton, Jatinder Grewal, Rod Hurford, Kylie Jacobs, Nermin Karamujic, Samantha Keogh, Kiran Shekar, Aashish Kumar, Vijo Kuruvilla, Kevin B. Laupland, Jayshree Lavana, Paula Lister, Stephen Luke, Langa Lutshaba, Josephine Mackay, Andrea Marshall, Prashanti Marella, Vikram Masurkar, Craig McDonald, James McCullough, Jason Meyer, Lynette Morrison, Lauren Murray, Ahmad Nasser, Ben Nash, Alex Nesbitt, Anni Paasilahti, Dinesh Parmar, Kunjal Patel, Hamish Pollock, Jaco Poggenpoel, Raju Pusapati, Lachlan Quick, Eamon Raith, Mahesh Ramanan, Gul E Rana, Josephine Reoch, Sunil Sane, Siva Senthuran, Vikram Shah, Judy Smith, David Stewart, Alexis Tabah, Cath Tacon, Jennifer Taylor, Mandy Tallott, Zephanie Tyack, Karthik Venkatesh, Adam Visser, James Walsham, Monica Watson, Stephen Whebell, Kyle White, Emma Williams, Patrick Young.

## Data availability

Deidentified data that support the findings of this study are available from the corresponding author on reasonable request, subject to Institutional Ethics Committee approval and data-sharing agreements.

## Funding

The authors gratefully acknowledge the QUT Centre for Healthcare Transformation for funding support.

## Conflict of interest

The authors declare the following financial interests/personal relationships, which may be considered as potential competing interests: Samantha Keogh reports a relationship with Becton Dickinson that includes consulting or advisory. Amanda Corley reports a relationship with Terumo Asia Holdings Pte. Ltd that includes funding grants. Amanda Corley reports a relationship with BioLife Solutions Inc that includes funding grants. Amanda Corley reports a relationship with Solventum Corporation that includes funding grants. Amanda Corley reports a relationship with Eloquest Healthcare Inc that includes funding grants. Amanda Corley reports a relationship with Wolters Kluwer that includes consulting or advisory. If there are other authors, they declare that they have no known competing financial interests or personal relationships that could have appeared to influence the work reported in this paper.
