# Supplementary material for: Research priorities for intensive care: A stakeholder-informed priority-setting partnership
Source: Crit Care Resusc. 2026 Sep 11;28(4):100215. doi: 10.1016/j.ccrj.2026.100215 (PMC13586683; doi:10.1016/j.ccrj.2026.100215)
Supplement: Supplementary file 1 [file mmc1.docx]

**SUPPLEMENTARY FILE**

**Supplementary File 1: Shortlisted research questions for interim prioritisation**

| **Theme** | **Sub-Theme** | **Question** |
| --- | --- | --- |
| Recovery and survivorship | Early mobilisation and preventing physical deconditioning | What strategies during ICU stay best prevent or minimise physical deconditioning and loss of function in critically ill adults while in ICU? |
|  | Rehabilitation after hospital discharge | What rehabilitation programmes and supports after hospital discharge best improve long-term recovery for ICU survivors? |
|  | Follow-up services and support for ICU survivors and families | What follow-up services and models of care best support recovery and wellbeing for ICU survivors and their families? |
|  | Long-term outcomes after critical illness | What are the long-term physical, cognitive and quality-of-life outcomes after critical illness, and how can these be prevented or improved? |
| Communication, partnership and shared decisions | Communication in the ICU | What communication approaches best support ICU patients and families during critical illness? |
|  | Shared decision-making and goals of care | How can shared decision-making and goals-of-care discussions be best supported in the ICU? |
|  | Consumer partnership and experience | How can consumer partnership, engagement, and co-design be used to improve the experience and delivery of intensive care services? |
| Workforce, wellbeing and culture | Staff wellbeing interventions | What interventions best support ICU staff wellbeing and psychological safety? |
|  | Impact of staff wellbeing on patient care | How does staff wellbeing and burnout affect patient safety and outcomes in the ICU? |
|  | Workforce culture, leadership and retention | What workforce and leadership strategies best support high-performing and sustainable ICU teams? |
|  | Workforce models | How should workforce roles and scope of practice across the multidisciplinary ICU team be optimised to improve patient outcomes and healthcare sustainability? |
| Brain health, sleep and delirium | Delirium prevention, management and sleep | What strategies best prevent and manage delirium and sleep disruption in critically ill adults while maintaining patient safety? |
|  | Starting and stopping ICU interventions | How should clinicians decide when to start, continue or stop ICU treatments and life-sustaining therapies? |
| Clinical decision making and monitoring | Using monitoring and tests to guide ICU decisions | How should monitoring, tests and imaging be used to guide treatment decisions and avoid unnecessary harm? |
| Resuscitation and precision care | Personalised haemodynamic targets | How should fluids, vasopressors and blood-pressure targets be individualised in critical illness? |
|  | Precision medicine in sepsis and shock | How can biomarkers and clinical features guide targeted therapies in sepsis and shock? |
| Airway management & respiratory support | Airway and emergent intubation safety | How can airway management and emergent intubation be made safer in the intensive care unit? |
|  | Delivery of respiratory support | What strategies best optimise the delivery and tolerance of respiratory support in critically ill adults? |
| Nutrition, metabolism and recovery | Nutrition delivery strategies | What nutrition support strategies (including timing, route and monitoring of feeding) best improve outcomes in critically ill adults? |
|  | Metabolic and muscle-preserving strategies | What metabolic and muscle-preserving therapies best prevent ICU-acquired weakness and improve recovery after critical illness? |
| Neurological care | Neurological care and prognosis | What strategies best optimise neurological care, prognosis, and recovery for critically ill patients with acute brain injury or hypoxic events? |
| Infection prevention and medications | Infection and antimicrobial resistance | What infection screening strategies best improve outcomes and antimicrobial stewardship in intensive care? |
| Medication continuity | Medication continuation | How should the continuation of patients’ pre-existing medications be managed during critical illness to improve recovery and prevent complications? |
| Early supportive therapies | Early supportive therapies | What early supportive therapies (such as dysphagia management) best improve outcomes for critically ill adults? |
| Advanced and specialised therapies | Advanced life support (e.g. ECPR/ECMO) | How should advanced life-support technologies (such as ECMO and ECPR) be used to improve survival and meaningful recovery in critical illness? |
|  | Renal and metabolic therapies | What renal and metabolic support strategies (including bicarbonate therapy and optimisation of renal replacement therapy) best improve outcomes in critically ill adults? |
| Systems, safety and innovation | Burden and harm of ICU care | What are the short- and long-term harms associated with ICU treatments, monitoring and devices, and how can avoidable harm be reduced? |
|  | Digital health/AI to improve ICU workflow | How can digital technologies and decision-support tools (including artificial intelligence) be used safely and effectively to improve care and workflow in intensive care? |
|  | Implementation Science in the ICU | What strategies best support the implementation of evidence-based practices in intensive care? |
| Ethics and decision making | Ethics and decision making | How do we make fair, appropriate, person-centred decisions about ICU care when outcomes are uncertain? |

**Supplementary File 2:** Ranking and scoring of shortlisted research questions from interim prioritisation survey responses

| Question | Rank | Score |
| --- | --- | --- |
| How can digital technologies and decision-support tools (including artificial intelligence) be used safely and effectively to improve care and workflow in intensive care? | 1 | 253 |
| How does staff wellbeing and burnout affect patient safety and outcomes in ICU? | 2 | 234 |
| What are the long-term physical, cognitive and quality-of-life outcomes after critical illness, and how can these be prevented or improved? | 3 | 229 |
| How should clinicians decide when to start, continue or stop ICU treatments and life-sustaining therapies? | 4 | 228 |
| What interventions best support ICU staff wellbeing and psychological safety? | 5 | 225 |
| What workforce and leadership strategies best support high-performing and sustainable ICU teams? | 6 | 224 |
| How should fluids, vasopressors and blood-pressure targets be individualised in critical illness? | 7 | 224 |
| What strategies during ICU stay best prevent or minimise physical deconditioning and loss of function in critically ill adults while in ICU? | 8 | 208 |
| What strategies best prevent and manage delirium and sleep disruption in critically ill adults while maintaining patient safety? | 9 | 206 |
| What strategies best optimise neurological care, prognosis, and recovery for critically ill patients with acute brain injury or hypoxic events? | 10 | 179 |
| How do we make fair, appropriate, person-centred decisions about ICU care when outcomes are uncertain? | 11 | 177 |
| How can biomarkers and clinical features guide targeted therapies in sepsis and shock? | 12 | 175 |
| What metabolic and muscle-preserving therapies best prevent ICU-acquired weakness and improve recovery after critical illness? | 13 | 175 |
| How can shared decision-making and goals-of-care discussions be best supported in ICU? | 14 | 165 |
| What follow-up services and models of care best support recovery and wellbeing for ICU survivors and their families? | 15 | 160 |
| What infection screening strategies best improve outcomes and antimicrobial stewardship in intensive care? | 16 | 160 |
| How should workforce roles and scope of practice across the multidisciplinary ICU team be optimised to improve patient outcomes and healthcare sustainability? | 17 | 154 |
| What are the short- and long-term harms associated with ICU treatments, monitoring and devices, and how can avoidable harm be reduced? | 18 | 152 |
| What rehabilitation programmes and supports after hospital discharge best improve long-term recovery for ICU survivors? | 19 | 145 |
| How can airway management and emergent intubation be made safer in intensive care? | 20 | 140 |
| What strategies best support the implementation of evidence-based practices in intensive care? | 21 | 140 |
| What communication approaches best support ICU patients and families during critical illness? | 22 | 135 |
| What strategies best optimise the delivery and tolerance of respiratory support in critically ill adults? | 23 | 124 |
| How should advanced life-support technologies (such as ECMO and ECPR) be used to improve survival and meaningful recovery in critical illness? | 24 | 106 |
| How should monitoring, tests and imaging be used to guide treatment decisions and avoid unnecessary harm? | 25 | 103 |
| How can consumer partnership, engagement, and co-design be used to improve the experience and delivery of intensive care services? | 26 | 92 |
| How should continuation of patients pre-existing medications be managed during critical illness to improve recovery and prevent complications? | 27 | 88 |
| What nutrition support strategies (including timing, route and monitoring of feeding) best improve outcomes in critically ill adults? | 28 | 86 |
| What renal and metabolic support strategies (including bicarbonate therapy and optimisation of renal replacement therapy) best improve outcomes in critically ill adults? | 29 | 83 |
| What early supportive therapies (such as dysphagia management) best improve outcomes for critically ill adults? | 30 | 40 |

**Supplementary File 3:** Content analysis of strategies to enhance research participation

| Theme | Quotes |
| --- | --- |
| Communication and Awareness | - Advertise the studies in the unit, get staff to get more familiar with the research studies making it easy for pts to be recruited - Weekly research meetings/ reviews or having one ward round a week that includes consideration of research projects  - Just being more visible and present - Good communication is key with everything I guess! |
| Facilitating Ease of Research Participation and Conduct | - Simplify or preferably automate recruitment processes - Less difficult ethics and governance pathways - Electronic data collection - Make the ethics application process more streamlined and less time consuming |
| Consumer Engagement | - Ensure information is provided in accessible formats, and that consumers are afforded choice around the best way to deliver information - Being human-centred is important - Engage family and patients on medical and surgical wards before they get sick and end up in ICU where may be too stressed to make contribution  - Broader range of consumer input |
| Organisational Support | - Protected research shifts - Provide protected rostering to perform research - Clinical support time - Dedicated research roles  - Encouraging management to support staff who engage in higher degrees |
